# Identifying prey capture events of a free-ranging marine predator using bio-logger data and deep learning

**DOI:** 10.1098/rsos.240271

**Published:** 2024-06-19

**Authors:** Stefan Schoombie, Lorène Jeantet, Marianna Chimienti, Grace J. Sutton, Pierre A. Pistorius, Emmanuel Dufourq, Andrew D. Lowther, W. Chris Oosthuizen

**Affiliations:** ^1^ Department of Statistical Sciences, Centre for Statistics in Ecology, Environment and Conservation (SEEC), University of Cape Town, Cape Town 7701, South Africa; ^2^ National Institute for Theoretical and Computational Sciences, South Africa; ^3^ African Institute for Mathematical Sciences, Cape Town 7945, South Africa; ^4^ Department of Mathematical Sciences, Stellenbosch University, Stellenbosch 7602, South Africa; ^5^ Centre D’Études Biologiques de Chizé, UMR7372 CNRS-La Rochelle, Villiers-en-Bois, France; ^6^ Department of Environment & Genetics, and Research Centre for Future Landscapes, La Trobe University, Melbourne, VIC 3086, Australia; ^7^ Marine Apex Predator Research Unit, Department of Zoology and Institute for Coastal and Marine Research, Nelson Mandela University, Gqeberha 6031, South Africa; ^8^ African Institute for Mathematical Sciences, Research and Innovation Centre, Kigali, Rwanda; ^9^ Norwegian Polar Institute, Tromsø, Norway

**Keywords:** accelerometer, animal behaviour classification, animal-borne video, chinstrap penguin, foraging ecology, machine learning

## Abstract

Marine predators are integral to the functioning of marine ecosystems, and their consumption requirements should be integrated into ecosystem-based management policies. However, estimating prey consumption in diving marine predators requires innovative methods as predator–prey interactions are rarely observable. We developed a novel method, validated by animal-borne video, that uses tri-axial acceleration and depth data to quantify prey capture rates in chinstrap penguins (*Pygoscelis antarctica*). These penguins are important consumers of Antarctic krill (*Euphausia superba*), a commercially harvested crustacean central to the Southern Ocean food web. We collected a large data set (*n* = 41 individuals) comprising overlapping video, accelerometer and depth data from foraging penguins. Prey captures were manually identified in videos, and those observations were used in supervised training of two deep learning neural networks (convolutional neural network (CNN) and V-Net). Although the CNN and V-Net architectures and input data pipelines differed, both trained models were able to predict prey captures from new acceleration and depth data (linear regression slope of predictions against video-observed prey captures = 1.13; *R* ^2^ ≈ 0.86). Our results illustrate that deep learning algorithms offer a means to process the large quantities of data generated by contemporary bio-logging sensors to robustly estimate prey capture events in diving marine predators.

## 1. Introduction

Large marine predators are important consumers that help maintain ecosystem functioning through top-down effects and recycling of limiting nutrients, among others [1,2]. Globally, marine predators consume millions of tonnes of prey annually, including prey species targeted by economically important commercial fisheries [3,4]. Therefore, predator consumption requirements are useful for fishery and conservation management policies (e.g. via the Natural Mortality parameter), especially where predators and fisheries display spatial and functional overlap [5]. Vast numbers of marine predators occur in the Antarctic Peninsula region of the Southern Ocean, especially during the austral summer. Baleen whales, seals, seabirds and other less conspicuous predators, such as finfish, are major consumers of lower trophic level prey, particularly Antarctic krill (*Euphausia superba*), a keystone species that is also targeted by a commercial fishery. Spatially explicit estimates of predator consumption can help identify potential risks that the fishery may pose to krill predators and can be used to inform policy decisions [6,7]. To date, estimates of predator consumption in the Antarctic Peninsula have typically been derived from bioenergetic models reliant on assumptions of field metabolic rates and prey energy content, a process that requires detailed data and often lacks empirical validation. Recently, it has become possible to estimate prey consumption more reliably using animal-borne tags (bio-loggers) equipped with high-resolution sensors [8]. Baleen whales, for example, are now thought to consume three times more prey than previously estimated [9]. Such findings have important implications for our understanding of trophic energy transfer, predator consumption requirements, species interactions and ecosystem-based fisheries management.

Penguins are important consumers in the Southern Ocean [3], with three species of *Pygocelis* penguins inhabiting the Antarctic Peninsula region: the Adélie (*P. adeliae*), gentoo (*P. papua*) and chinstrap penguins (*P. antarctica*). Here, Antarctic krill is a major food source for all three *Pygocelis* penguins, but chinstrap penguins have a particularly narrow trophic niche dominated by krill consumption [10]. While chinstrap penguins are still one of the most abundant krill-dependent predators breeding in the Antarctic Peninsula region, population declines greater than 50% have occurred through large parts of their range [11,12]. These declines may be linked to reduced food availability through climate-induced habitat modification or competition with other predators, including the krill fishery [11–13]. Despite this, there are currently no validated methods available to directly measure prey capture rates of krill-feeding penguins. This information is fundamental for monitoring their functional responses (i.e. the relationship between predator feeding rates and prey abundance [14]) and linking penguin population dynamics with estimates of prey consumption rates.

Early attempts to identify prey capture rates in wild penguins were intrusive (e.g. oesophageal temperature sensors and beak-angle sensors) and had high device failure rates, resulting in low sample sizes [15–18]. Other studies have used undulations in dive profiles to estimate prey capture rates in foraging penguins. While undulations may correlate with prey captures in some piscivorous penguin species [15,16,18], it appears harder to quantify prey ingestion rates in chinstrap and other krill-feeding penguins with dive metrics [19]. More recently, animal-borne video cameras have been used to identify prey types, prey capture events (PCE) and foraging interactions in a variety of penguin species [8,20,21]. Video footage can provide direct evidence of prey ingestion (rather than relying on inferences [22,23]) but recording duration is typically only several hours and their data quality is dependent on ambient light conditions, as well as water clarity. Video loggers are thus well suited to provide a ‘gold standard’ of prey capture rates for short periods, under certain conditions, but other methods are needed to estimate prey consumption throughout foraging trips.

Tri-axial accelerometers measure instantaneous acceleration at very high sampling frequencies (usually up to 100 Hz in bio-logging studies). When attached to animals, accelerometer-derived data can be used to infer body angles and behaviours [24]. Accelerometer loggers record data over much longer periods than video cameras (days compared with hours when using comparable power sources) and their data files are several orders of magnitude smaller. Thus, accelerometers are a compelling tool for studying the behaviour of free-ranging animals [25–27]. The use of back-mounted accelerometers is particularly appealing for seabirds, as the loggers are small, quick to deploy and non-invasive. Accelerometer data have been used to detect prey captures in several diving seabirds, especially in piscivorous species where prey-handling times can last several seconds, producing clear accelerometer signals [28–31]. Penguins feeding on smaller prey, such as krill, can consume multiple prey within a second, and high data sampling rates (possibly from multiple accelerometers) are needed to accurately describe prey captures [8,32].

Machine learning is well-suited to rapidly process the large amount of data generated by high-throughput bio-logging tags such as accelerometers, allowing classification of multiple data streams at rates not possible through manual classification. Commonly used machine learning algorithms in bio-logging science include Random Forest (RF) and Support Vector Machine (SVM) algorithms [30,33]. These algorithms generally rely on a multitude of user-determined statistical variables, a process that can be laborious and time-consuming to apply. Neural networks can outperform simpler machine learning methods [34] and deep learning (neural networks with multiple hidden layers) has become more prevalent in marine science studies in recent years [35]. A deep learning application to quantify marine predator consumption rates therefore has great potential to increase our understanding of their foraging ecology, and to provide data and analytical tools useful for ecosystem management.

The aim of this article is to develop an analysis pipeline to detect individual prey captures of Antarctic krill by wild chinstrap penguins. We use penguin-borne videos to validate prey captures, and supervised learning based on neural network architectures to train on accelerometer and depth data. Specifically, we tested how well two existing deep learning architectures—a convolutional neural network (CNN) developed for human activity recognition [36], and V-Net adapted to time-series data [37]—could be trained on accelerometer data, to produce models that effectively predict PCE. Our ultimate goal is to produce a workflow that can identify PCE from new acceleration and depth data. This will enable future practitioners to quantify spatio-temporal patterns of prey consumption, information applicable to ecosystem monitoring and management.

## 2. Methods

We collected data from chinstrap penguins breeding at Monroe Island (January 2022; *n* = 42 deployments) and Powell Island (January 2023; *n* = 54 deployments) in the South Orkney Islands (60.6° S, 45.6° W). Customized video loggers (Zoolog Solutions) and dive-accelerometer loggers (Axy-Trek Marine or AGM; TechnoSmArt) were deployed, in tandem, on birds with chick(s) and a partner present at the nest. The video loggers recorded high-definition (720 p) videos at 30 frames per second, saved as video files of 5–15 min in an AVI or MOV format. To facilitate synchronization of time between different loggers (see §2.2 below), a short (10 s) video was recorded at the time of deployment, after which the logger switched to standby mode until a programmable delay time had elapsed. The video logger then recorded continuously, typically for 3–4 h when the battery depleted. The Axy-Trek and AGM loggers recorded tri-axial accelerometer data at 25 Hz, and temperature and depth (TDR) data at 1 Hz typically lasting a full foraging trip. The loggers were attached to the feathers along the mid-point of the back (on the median plane, with the camera immediately anterior to the accelerometer logger) using Tesa tape and Loctite glue [38]. During both deployment and retrieval, the penguin was caught by hand, blindfolded with a soft cloth hood and restrained (<5 min) while the loggers were deployed or retrieved. We aimed to retrieve the loggers after a single foraging trip.

### 2.1. Analysis

**Figure 1.**
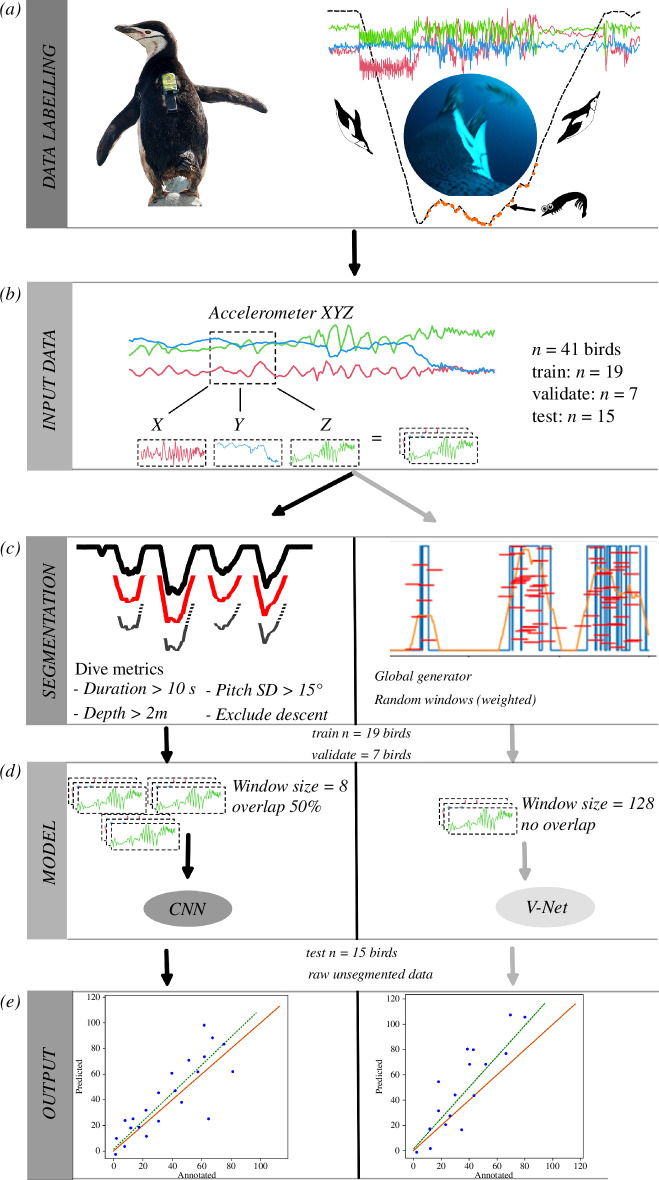
Workflow from data acquisition to model output. (*a*) Bio-logging data (tri-axial acceleration, depth and video) are time synchronized and labelled where PCE are observed in the video. (*b*) The input data used in the models are the three accelerometer axes, which are (*c*) balanced through dive thresholds for CNN and using a global generator (fig. 3 in [37]) with random sampling windows for V-Net. (*d*) The data are fed into the CNN model in windows of eight data points with 50% overlap, and windows of 128 data points with no overlap for V-Net. (*e*) The trained models are used to predict PCE for 15 individuals (not used during training) and predicted PCE are regressed against the labelled PCE for each dive to assess model performance.

The analysis workflow, illustrated in figure 1 and detailed below, involved several steps. Initially, we synchronized video and accelerometer–TDR data and identified PCE in the video (figure 1*a*). Subsequently, individual bird data were divided into training, validation and test groups, with efforts to balance prey capture and non-prey capture labels (figure 1*b*). The CNN training and validation data were balanced by selecting subsets based on dive parameters, while the V-Net utilized weighted random selection of windows (figure 1*c*). Both models were trained using short segments of data from the three accelerometer axes (figure 1*d*). The models predicted the number of PCE per dive for the ‘test’ data set (penguins not part of training or validation), and we compared these predictions with the number of observed PCE in the video (figure 1*e*). Specifically, predictions of the number of PCE were restricted to periods where the penguin was diving (depth > 0.4 m) to exclude spurious predictions at the water surface.

### 2.2. Video labelling

Some animal-borne video cameras use frame duplication (where copies of frames are inserted into the video as additional frames) to achieve artificially high frame rates. Frame duplication can create problems for synchronization of different data streams. We, therefore, removed duplicated frames (changing the frame rate from 30 to 24 frames per second) while converting individual video clips to MP4 format using the *ffmpeg* software (ffmpeg.org). The video and accelerometer data were then synchronized by finding distinct, high-energy behaviours that were visually evident in both data streams. These behaviours include shaking of the accelerometer loggers at deployment (during the 10 s video) and at-sea events such as the penguin shaking its feathers, repeated side-to-side rolling during bathing and exiting the water after a dive (see electronic supplementary material, figure S1). During synchronization, the accelerometer time-series data was taken as the ‘correct’ time, and the video was then matched to the accelerometer time-series, to determine the temporal offset. Synchronization of video and accelerometer data was done for every video clip to account for any clock drift, and to ensure temporal accuracy as close as possible to the sampling rate (25 Hz). We used VANTAGE, a customized interface written in Python (available at github.com/sschoombie/VANTAGE) to synchronize video and accelerometer data, and to view and annotate PCE in videos. Prey capture events were therefore identified through visual inspection of videos that were time-synchronized with accelerometer data, and accordingly annotated on both data streams (figure 1*a*).

Three observers annotated videos, each labelling a separate data set. A small subset of videos was labelled by all three observers to assess potential observer effects, but there was good agreement between observers. Chinstrap penguins predominantly target Antarctic krill, a comparatively small and slow-moving prey species that is consumed individually with minimal handling times per prey capture. We labelled PCE as instances where krill was clearly observed to be captured. For each PCE a window of eight data points was labelled (three before and four after the moment of capture) as most (~95%) of PCE were > 0.32 s (or eight data points at 25 Hz) apart (see electronic supplementary material, figure S2). This was done to ensure that a short sequence of accelerometer data (instead of single data points) was associated with each PCE, without the risk of merging data from consecutive PCE. Where video quality was too low to identify individual krill, deliberate head strikes were also assumed to be PCE [19,22]. We did not distinguish between successful and unsuccessful PCE as preliminary analyses showed that nearly all prey capture attempts were successful [19,39]. Ambient light became a limiting factor when penguins were diving deep, especially in overcast or low solar angle conditions. As such, video sequences that were too dark for relevant features (i.e. head strikes) to be detected were excluded from the analysis.

### 2.3. Preparation of depth data

Depth data (1 Hz) were linearly interpolated to match the 25 Hz sample rate of the accelerometer data. We defined dives as any depth greater than 0.4 m, and identified the start and end point of each dive as the point where the penguin crossed this threshold. Dives with a maximum depth ≤ 2 m or a duration ≤ 10 s were classified as ‘surface dives’ as they did not have enough data to split into dive phases; deeper and longer dives were split into three phases (descent, bottom and ascent) based on the maximum depth and depth rate of change. The descent phase started at the first point of the dive and ended where the normalized depth rate of change was <0.5, and the depth was >50% of the maximum depth (similar to [40]). The ascent phase was the inverse of descent, and the bottom phase was the period between descent and ascent. Any dive where at least one PCE was observed (through video labelling) was regarded as a ‘foraging dive’.

### 2.4. Machine learning models

We compared how well two deep neural networks were able to predict PCE during dives (defined above) using normalized tri-axial accelerometer data (*X* - surge, *Y* - sway and *Z* - heave axes). Our first approach is based on a simple CNN developed for human activity recognition [36]. Second, we use a more complex fully connected convolutional network (V-Net) developed for three-dimensional image analysis [41] and customized for behavioural classification from accelerometer data [37]. Convolution can be likened to a specialized filter that scans over an input, looking for specific patterns. This process aids the network in identifying features within the data by conducting mathematical operations sequentially on small segments of the input [35]. When behaviours are derived from time-series data (e.g. accelerometer data), these sequences are autocorrelated. The convolution mechanism is well suited to classify such data by learning from local patterns in the data while also accounting for variation in the scale of patterns [36].

The two neural networks were trained and evaluated by randomly assigning individuals to training, validation (hyperparameter tuning) and testing groups. With every random assignment of individuals, we ensured that 50%, 20% and 30% of the annotated PCE were retained in each group. This step was necessary as some individuals had very few PCE and if these individuals were overrepresented in the training data there were not enough data for the model to learn from. This process of random selection of individuals and model training/testing was repeated 10 times, and the split of individuals with the best prediction performance (regression slope and line fit closest to 1 for both models, see §2.6 below) was retained.

The CNN was trained with segments of data from each of the three accelerometer axes (*X*, *Y* and *Z*; figure 1*b*), with a window size of eight data points (*w*_CNN_ = 8) and 50% overlap between windows, resulting in a 8 x 3 matrix (figure 1*d*). These data segments were sampled for each dive separately and only periods of ‘most likely foraging’ (see §2.5 below) were used. Each segment had a single label associated with it, where 1 represented a PCE and 0 no PCE. The model had two 1D convolutional layers, sliding according to the time dimension, with 128 filters, a kernel size of 2 and a rectified linear unit (ReLU) activation function to capture local patterns (or features) in the input sequence. This was followed by a dropout layer with a rate of 0.2 (to prevent overfitting), and a pooling layer to reduce the spatial dimensions of the feature maps by a factor of 2, while retaining the most important information. Finally, the one-dimensional feature maps generated by the convolutional layers were flattened to a one-dimensional vector and passed through a fully connected (dense) layer with 100 units and a ReLU activation function, before being passed through the output layer where the input segment is labelled with a prediction for each of the two categories (i.e. PCE or no PCE). The final layer used the *softmax* activation function, which converted the network’s raw output into class probabilities. The final predicted probabilities were then converted to a binary classification by retaining the class with the highest predicted probability (1 for PCE and 0 for no PCE). The training process consisted of 10 epochs with all the data passed through the training data set in batches of six segments at a time, updating the model’s internal parameters after each batch. Over the course of these 10 epochs, we used the popular Adam algorithm to optimize the weights of the model and compared the obtained predictions to the labels using a categorical cross-entropy loss on the validation data set after each epoch. The values of these hyper-parameters were optimized by rerunning the models with varying values and retaining the values where the loss was lowest.

The V-Net model offers the benefit of reconstructing the input signal with the predicted label instead of the input values, i.e. the surrounding points are taken into consideration during prediction [41]. This allowed us to generate predictions for each data entry, or time point, of the recorded data, eliminating the need to unpack labelled segments (or windows) of data. We provided the V-Net algorithm with the same three accelerometer axes as above, but a larger window of data was used at a time (*w*_V-Net_ = 128 data points, or 5.12 s of data), resulting in a 128 × 3 matrix as input. To train the V-Net model, the windows were randomly drawn with a bias on the entire training data set (see §2.5 below). Then a sequence of one-dimensional convolutions (based on the time dimension) was performed on the input data. The initial layers in these successive sequences, referred to as the encoder, are designed to compress the signal and generate diverse features. Meanwhile, the latter part of the network, known as the decoder, is responsible for synthesizing these generated features to predict the outcome (1 for PCE or 0 for no PCE), all while reconstructing the signal. The detailed architecture of the V-Net for time series can be found in [37]. To train the V-Net, we used the Adam optimizer and utilized the generalized dice loss function as proposed by Milletari *et al*. [41]. The generalized dice loss is a metric used in image segmentation to handle class imbalance by incorporating class weights. The training process consisted of 10 epochs, each involving a pass through the training data set, comprising 20 000 windows fed to the optimizer in batches of 32. After each epoch, we evaluated the loss using 16 000 windows from a separate validation data set. Over the 10 epochs, we retained the weights corresponding to the epoch with the lowest validation loss.

### 2.5. Data balancing

Our data set was highly imbalanced, with PCE labels accounting for only 2% of the raw data (i.e. all annotated data including surface and diving behaviour). Convolutional networks, like most machine learning algorithms, are sensitive to imbalanced data [42], and we therefore had to address this problem to improve the performance and accuracy of both our models. We used two different approaches for the CNN and V-Net model (figure 1*c*). When training the CNN, we only used data from the most plausible foraging periods, i.e. where most of the PCE were observed while labelling the videos. Krill-eating penguins typically forage during deeper dives and display limited foraging during the descent phase of a dive [19]. Penguins also display dynamic body movement during foraging, which results in a larger range of pitch angles (rotations around the heave axis). Therefore, larger values of the standard deviation of pitch are typically associated with foraging dives [30]. To obtain pitch, we first calculated static acceleration (*X*_s_, *Y*_s_ and *Z*_s_) using a 2 s running mean across the raw accelerometer data (*X*, *Y* and *Z*). From these values, the pitch angles were calculated following the equation in [43] so that


$$pitch\;=\;atan2\left(X_{s},\sqrt{Y_{s}^{2}+\;Z_{s}^{2}}\right),$$

which was subsequently converted from radians to degrees. Exploratory analysis of the labelled accelerometer-depth data set showed that 93% of the observed PCE could be retained while reducing the size of the data set by 77% by using the following thresholds: dive maximum depth > 2 m, dive duration > 10 s, dive pitch standard deviation (s.d.) > 15° and exclusion of descent phase (see electronic supplementary material, figures S3–S5). Thus, by removing areas of unlikely foraging, we could produce a more balanced data set (~6% representing PCE) that was used to train the CNN.

Implementation of the V-Net model, as described in [37], already accounts for imbalanced data through its dice loss function [41] as well as the use of a customized global generator. This generator selects windows of data from the training data set in a random manner, with a deliberate bias towards PCE data (figure 1*c*). Specifically, 10% of the windows were randomly chosen from various individuals within the training data set, while the remaining 90% were drawn with a higher probability of encompassing PCE occurrences within the window (see [37] for details).

### 2.6. Model evaluation

We regressed the total predicted PCE against the total annotated PCE within individual dives to assess how accurately the CNN and V-Net models predicted PCE in the testing data set (i.e. data that have not been used in the training process) (figure 1*d*). It is important to stress that evaluation took place at the dive level, and that it considered all video-observed PCE within dives (i.e. depth > 0.4 m). When training the model, a single PCE was labelled as a window of eight data points (see §2.3); thus, when testing the model prediction, the total number of data points predicted as PCE was divided by eight to estimate the total number of PCE in the dive. Predictions at the surface (i.e. depth < 0.4 m) were ignored, although some annotated PCE (*n* = 46, 0.2% of total) were observed to occur here (not to be confused with ‘surface dives’, that occurred between 0.4 m and 2 m) (see §3). The slope, intercept and coefficient of determination (*R* ^2^) obtained from the observed vs. predicted linear regression were compared with a 1:1 regression, and to previous studies that estimated PCE from penguin-borne bio-loggers [16,18,31].

Three performance metrics (accuracy, sensitivity and false-positive rate) previously used to evaluate machine learning models for penguin PCE [30,32] were calculated. Accuracy is the proportion of correct predictions made by the model, sensitivity is the number of correctly predicted PCE (true positive) divided by all predicted PCE (true positive and false positive) and the false positive rate is the amount of incorrectly predicted PCE (false positive) divided by the total observed PCE. These metrics were calculated at the dive level, i.e. to differentiate between foraging dives (with at least one PCE) and non-foraging dives (no PCE), as well as for individual data points, i.e. to compare the outcome of each point (PCE or no PCE). However, because we could not ensure exact matching of times between video and accelerometer data, we smoothed the data to 1 s when calculating the point-wise performance metrics. All data analysis was performed in the Python programming language (v. 3.11.5) with model training implemented in Tensorflow 2 [44] using a laptop with an AMD Ryzen 9 5900HX 4.60 GHz processor, 32 GB internal RAM and a NVIDIA GeForce RTX 3070 (8 GB RAM) graphics processing unit. Values are means ± s.d. unless otherwise stated.

## 3. Results

We retrieved loggers from all deployments (*n* = 96), but not all deployments yielded data useful for this study. The full complement of data (overlapping video, accelerometer and TDR data) were obtained for 28 deployments in 2022, and 45 in 2023. However, low ambient light in the bottom phase of the dives in 2023 reduced PCE annotation to 13 individuals. Thus, data from 41 individuals collected over two breeding seasons were used in the analyses. In total, 151 h of video footage were recorded during which time penguins made 3184 dives (~59 h). Of these, 1606 were foraging dives (~14 h) during which approximately 23 000 PCE were recorded (see electronic supplementary material, video S1 for examples of observed prey captures). Most of the observed prey captures occurred at depths deeper than 0.4 m, but a small number (46 observations; 0.2% of total PCE) occurred shallower than 0.4 m. These surface PCE were not considered during our analyses. The video footage was not clear enough to identify krill species with confidence, but most krill appeared to be Antarctic krill. Penguins sometimes captured distinctly smaller euphausiids (potentially juvenile Antarctic krill or other krill species) and on rare occasions, individuals also appeared to consume gelatinous prey, but we did not differentiate between prey types.

**Figure 2.**
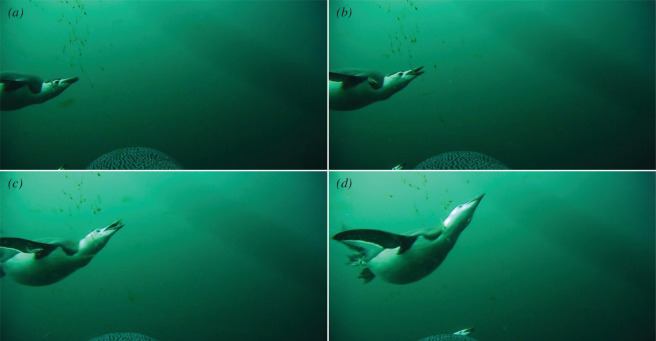
A sequence of frames (*a–d*) lasting 0.16 s shows a chinstrap penguin catching an individual krill from a small swarm.

**Figure 3.**
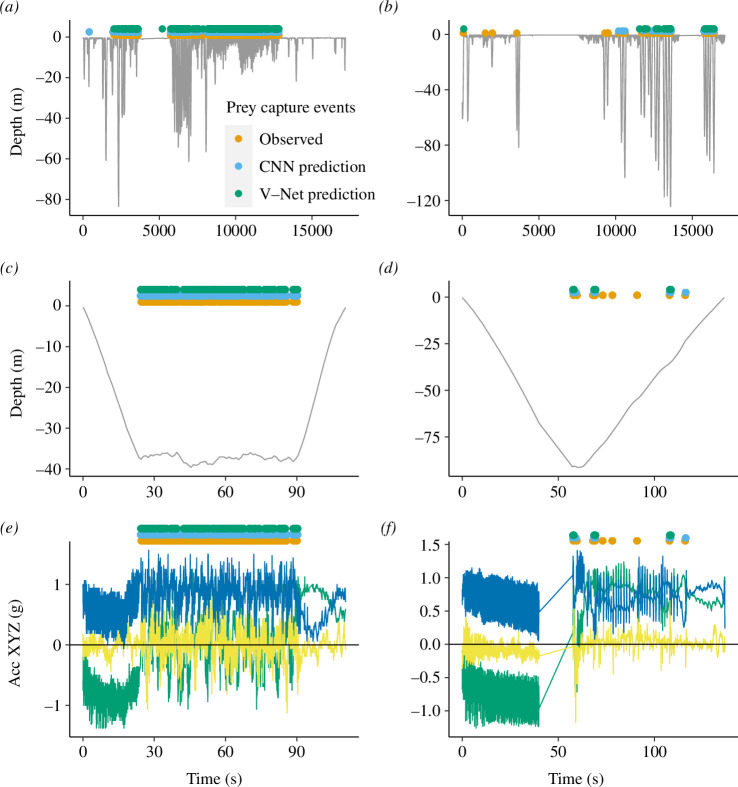
Example of labelled and predicted PCE from two chinstrap penguins, showing high-density foraging (*a,c,e*) and low-density foraging (*b,d,f*), respectively. The total labelled data set (*a,b*) as well as single dives (*c,d*) show diving data (grey), labelled PCE (orange) and predicted PCE (blue and green). Accelerometer traces (*e,f*) for the individual dives are also shown. The gap in data in (*f*) is an area where no video was available owing to low ambient light**—**the data for this part of the dive were removed for the analysis.

The number of observed PCE varied substantially between individuals, with four birds being at sea but not foraging at all within the video recording period. These four individuals were not considered when dividing the data for model training (i.e. only 37 individuals were considered), but they were included when testing the model performance. We used 19 individuals for training, 7 for validation during training and 15 individuals for testing. Training took approximately 1 min per epoch for the CNN and V-Net, respectively. Both the CNN and V-Net models were able to predict individual PCE from tri-axial accelerometer data, but additional information from depth loggers was necessary to exclude a high number of spurious predictions at the surface (i.e. not within dives). From the video analysis, it was apparent that the penguins often perform an upward striking motion when capturing krill (figure 2). The accelerometer data seemingly described this upward striking motion and areas of high-density foraging could be visually discerned at a broad scale (i.e. at a scale of several seconds). However, it was not possible to identify individual PCE by eye (figure 3).

**Figure 4.**
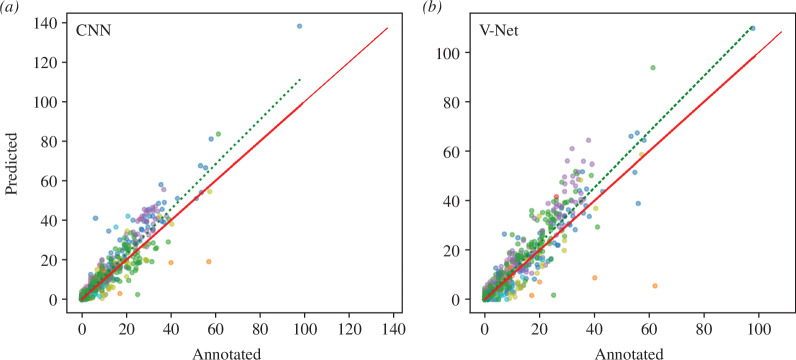
Predicted PCE from two deep learning models (*a*, CNN; *b*, V-Net) trained on tri-axial accelerometer data and validated with bird-borne video footage. The predictions are from 15 penguins that were not used in the training of the models. The different coloured points represent individual penguins. Green dashed lines show the linear regression of annotated and predicted PCE per individual dive, while the red solid line shows a 1:1 linear regression.

At a dive level, the CNN model misclassified 7% of non-foraging dives (when no PCE were observed in the video) as foraging dives, while 2% of V-Net dives were misclassified (false positives). However, when non-foraging dives were misclassified as foraging dives, these always had very low predicted PCE within the dive (mean = 1, s.d. = 1 and range 1–8). Both models had a low proportion (< 1%) of false negative foraging dives, i.e. where PCE were observed in the video, but no PCE were predicted to occur. Regression analysis of the best-performing models showed a near one-to-one linear relationship between observed PCE and predicted PCE (CNN slope = 1.13 and V-Net slope = 1.13). The coefficient of determination was high for both models (CNN *R* ^2^ = 0.86, V-Net *R* ^2^ = 0.87) indicating relatively little variation around the regression line (figure 4).

**Figure 5.**
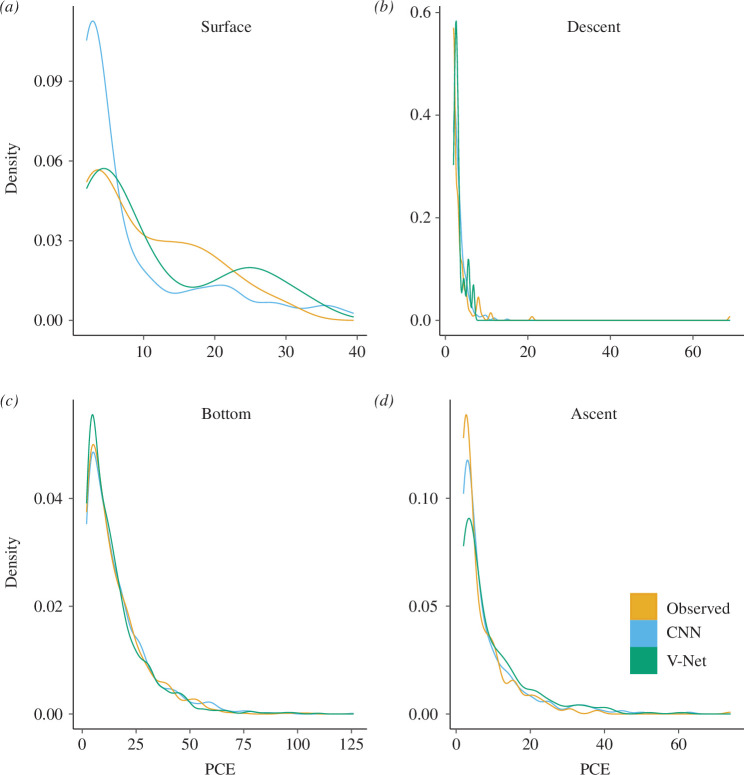
Kernel density plots showing the distribution of PCE per dive, as observed from video loggers attached to chinstrap penguins and predicted by two deep learning models (CNN and V-Net). Separate plots are shown for the different dive phases: (*a*) surface behaviour, (*b*) descent phase, (*c*) bottom phase and (*d*) ascent phase.

On average, individual dives differed by 2.4 ± 3.7 (CNN) and 3.3 ± 4.6 (V-Net) PCE from video observed PCE when at least one PCE was observed in the video. Most (83%) of the video-observed PCE occurred in the bottom phase of dives, although a non-negligible proportion (12%) of PCE occurred in the ascent phase (figure 5). Our model predicted PCE corresponded well with these video annotation estimates (bottom phase: CNN = 74%; V-Net = 80%; ascent phase: CNN = 12%; V-Net = 15%). The four individuals for which we did not observe any PCE had predicted PCE in 9% (CNN) and 2% (V-Net) of dives, but the total predicted PCE for these dives were low (never exceeded three PCE per dive).

## 4. Discussion

This study presents a novel, validated method that uses acceleration and depth data to quantify krill capture rates by chinstrap penguins. We evaluated two deep neural network algorithms, CNN and V-Net, each requiring distinct expertise. The CNN required data pre-processing, and the V-Net a more thorough understanding of deep learning algorithms. Despite these differences, both algorithms produced comparable results when tested against PCE observed from penguin-borne video cameras and demonstrated ease of adaptation according to user preferences. The CNN was easier to implement, but the segmented nature of the input data limits its application to species with similar foraging habits. In contrast, the V-Net model was able to predict on individual data points and could potentially be used on species with varying scales of foraging patterns, such as Adélie and macaroni penguins *Eudyptes chrysolophus* that prey on both krill and fish [32]. Irrespective, our results illustrate that these deep learning algorithms are powerful tools capable of identifying PCE within individual dives with high precision and accuracy (slope = 1.13 and *R* ^2^ = 0.86 and 0.87 for CNN and V-Net, respectively). Broader application of the methods we developed here has potential to enhance our understanding of functional responses and consumption requirements of penguins in Antarctic waters.

### 4.1. Identifying PCE in penguins

Video observations proved to be a valuable tool to observe penguin PCE, even though a large portion of the video footage collected during our second field season was unusable owing to insufficient ambient light, a common problem in video analysis of marine animals [19,32,39]. In our case, ambient light became too low when penguins were diving deeply (> 70 m), where many of the PCE probably occurred but could not be labelled. Crepuscular and nocturnal hunting may further limit the utility of standard video cameras [45]. This clearly illustrates that methods other than video observations will often be needed to estimate prey consumption. Although video footage suffers from light and other limitations (e.g. comparatively short sampling durations and uncertainty about whether PCE occurred [19]), we were able to generate a comprehensive labelled data set for 41 individual chinstrap penguins. This sample far exceeds the amount of validation data available in previous studies that identified prey captures from accelerometers in other penguin species [30–32].

We correctly classified over 90% of dives as foraging or non-foraging, an improvement in accuracy over methods based on dive profiles alone [19]. The use of machine learning to classify accelerometer data into broad behavioural states has increased in recent years, but the few that identified fine-scale prey capture behaviour are often limited by small sample sizes [28,29,31]. We were able to quantify PCE within dives, and our analyses not only allowed us to identify foraging areas (i.e. foraging dives) but also to quantify the consumption of prey. Crucially, we were able to do this using back-mounted accelerometers, which are easier to deploy, less invasive and feasible for smaller species when compared with previous methods such as head- or bill-mounted loggers [28,30,31]. As expected, chinstrap penguins nearly exclusively preyed on krill**—**the only other prey observed captured was gelatinous species, but these were very rare observations. Other krill-eating penguins are known to consume carnivorous gelatinous prey, but at a low level, and almost never herbivorous salps [20,23]. In the absence of adequate training data, we did not test whether the deep-learning models could differentiate between prey types. However, given the rarity of other prey types in the present study, it is unlikely to influence model performance or be biologically relevant. Separating krill, gelatinous and other prey types may be more important in other penguin species that have broader trophic niches. For example, macaroni penguins prey on a mixture of krill and small fish, and where machine learning has been used to identify their capture rates, a depth threshold was used to distinguish probable prey types [32]. We rarely observed unsuccessful prey capture attempts (where the penguin pursues prey but does not catch it), and therefore did not differentiate between successful and unsuccessful captures. Distinguishing between actual and attempted prey captures would be important to consider for predators with lower prey capture success rates [28].

### 4.2. Deep learning to identify prey captures in marine predators

High sampling rates are required to detect fine-scale events such as prey captures in bio-logging data. Indeed, for this study, more than 30 GB of accelerometer–TDR data, representing > 2700 h, were generated. Even if this volume of data could be manually analysed, it is challenging to visually identify PCE (that occurs at the sub-second level) in the accelerometer data. Few studies have developed machine learning algorithms to identify PCE in accelerometer data obtained from seabirds, and these studies have primarily focused on piscivorous penguins. Brisson-Curadeau *et al*. [31] used a neural network with one fully connected layer to study PCE in two king penguins (*Aptenodytes patagonicus*) but other studies used conventional machine learning methods like SVM or k-nearest neighbour [28,30,32,34]. These algorithms are based on the computation of numerous descriptive variables or ‘features’ (e.g. mean, s.d., kurtosis, skewness, etc., of a range of accelerometer metrics) [28,30,32]. One major advantage of deep learning models is that they do not need these descriptive variables, but that they are trained with raw accelerometer data [46]. This negates the time-consuming task of creating such features, which often also needs expert input to construct. Our deep learning models performed well on the raw data and, although some prior knowledge was needed to construct a balanced data set for the CNN, the global generator used in the V-Net model negated the need for a-priori knowledge about penguin foraging behaviour. Deep learning has rarely been used to analyse the foraging behaviour of penguins (e.g. [47] used deep learning in the context of computer vision problems of penguin-borne video) and other marine predators. Ngô *et al*. [48], for example, used accelerometer data and a U-Net architecture (similar to V-Net) to identify foraging events in narwhals (*Monodon monoceros*).

Deep learning architectures are now well documented and have thus become widely accessible. Our results show that these methods can accurately classify PCE from large bio-logging data sets. Although the CNN model architecture was comparatively simple, it required segmentation of the data into overlapping windows, but this is common practice in machine learning applications with many examples available [30,32,36]. The V-Net model was more complex to implement as it uses many customized functions, but these are also well documented in their original descriptions [37,41]. The CNN had more false positive dives and more PCE predictions during the descent phase, likely because the descent phase was excluded from the training data to better balance the training data set. The global generator used by the V-Net model seemed to be a more robust approach, where the training data still included the descent phase as well as periods at the surface. However, a drawback of the global generator was that it only used a sample of the data, and on rare occasions, if an ‘unlucky’ sample of data (e.g. disproportionate amount of low PCE dives) was chosen during training, the model accuracy dropped considerably. However, this was easily rectified by resampling with the global generator and rerunning the model.

Our work is based on supervised machine learning models, but the integration of supervised and unsupervised learning can, in some instances, further increase the robustness of outputs [36,49]. For example, in marine predators, predictions of PCE could be improved by first classifying broad foraging behaviour in an unsupervised manner, followed by supervised learning. Here, and in other studies where video loggers are deployed in conjunction with accelerometer-depth loggers, the latter usually have longer recording periods than the former. These ‘excess’ data that do not overlap with video (and are thus not used for supervised learning) can potentially be used for unsupervised pre-training, with supervised training being done where the data overlaps. Where training data is lacking, data augmentation may also be an effective tool to train deep learning models [50].

Further model evaluation is needed to determine whether behavioural plasticity or site-level variability may affect how well our model generalizes to predict PCE from other chinstrap penguin populations. In general, one should be cautious when making predictions from new data, as the effect of individual variability on the performance of most machine learning algorithms is still poorly understood [49]. That said, supervised models with large sample sizes tend to generalize well, allowing predictions at the population level and even across species [49]. Given our supervised learning approach and large sample size, we expect that our results should be scalable to the population level, allowing prediction of PCE for new individuals where only accelerometer and depth data are available.

### 4.3. Marine predator consumption and ecosystem management

Quantifying consumption by marine predators is vital to understand trophic energy transfer, which fundamentally influences population and community dynamics. While marine predator populations are declining in many systems owing to altered bottom-up control [51], other populations are recovering from earlier dramatic declines [52]. In both these scenarios, the ability to measure predator prey consumption is critical to understanding trophic relationships, to predict future population dynamics and to inform resource management strategies. For example, the monitoring of marine predator populations (e.g. breeding population sizes, breeding success and foraging performance) is an integral part of ecosystem-based fisheries management, to ensure the sustainability of both predator and harvested populations. In the Southern Ocean, numerous krill-dependent marine predators (including chinstrap penguins) are monitored as part of the Commission for the Conservation of Antarctic Marine Living Resources Ecosystem Monitoring Program (CEMP) [53]. In this context, improving our ability to quantify predator functional responses, and to estimate the spatial distribution and consumption of prey by krill-dependent predators are important goals. Modern bio-logging tags with depth and accelerometer sensors can record data over periods of days to weeks, and our analyses show that deep learning models can reliably detect PCE in this data, even in a predator that rapidly consumes small prey items. The advent of new technology and methods, including those developed here, could therefore contribute to validate or improve current estimates of prey consumption by marine predators, to enhance CEMP and other ecosystem monitoring programmes.

## Data Availability

Example data and scripts used in the analysis are available on the University of Cape Town’s ZivaHub repository [54]. Scripts associated to the V-Net architecture are available at [55]. Supplementary material is available online [56].
